# Early warning of *Aspergillus* contamination in maize by gas chromatography-ion mobility spectrometry

**DOI:** 10.3389/fmicb.2024.1470115

**Published:** 2024-09-26

**Authors:** Yucan Qin, Haoxin Lv, Yating Xiong, Lin Qi, Yanfei Li, Ying Xin, Yan Zhao

**Affiliations:** ^1^School of Food and Strategic Reserves, Henan University of Technology, Zhengzhou, China; ^2^China National Research Institute of Food and Fermentation Industries, Beijing, China; ^3^College of Food Science and Engineering, Henan University of Technology, Zhengzhou, China

**Keywords:** maize, gas chromatography-ion mobility spectrometry (GC-IMS), *Aspergillus flavus*, *Aspergillus niger*, volatile organic compounds (VOCs), principal component analysis (PCA)

## Abstract

**Introduction:**

As one of the main grain crops in China, maize is highly susceptible to *Aspergillus* infection during processing, storage and transportation due to high moisture at harvest, which results in the loss of quality. The aim of this study is to explore the early warning marker molecules when *Aspergillus* infects maize kernels.

**Methods:**

Firstly, strains MA and MB were isolated from moldy maize and identified by morphological characterization and 18S rRNA gene sequence analysis to be *Aspergillus flavus (A. flavus)* and *Aspergillus niger (A. niger)*. Next, fresh maize was moldy by contaminated with strains MA and MB. The volatile organic compounds (VOCs) during the contamination process of two fungal strains were analyzed by gas chromatography-ion mobility spectrometry (GC-IMS). A total of 31 VOCs were detected in maize contaminated with strain MA, a total of 32 VOCs were detected in maize contaminated with strain MB, including confirmed monomers and dimers. Finally, heat maps and principal component analysis (PCA) showed that VOCs produced in different growth stages of *Aspergillus* had great differences. Combined with the results of GC-IMS, total fungal colony counts and fungal spores, it was concluded that the *Aspergillus*-contaminated maize was in the early stage of mold at 18 h.

**Results:**

Therefore, the characteristic VOCs butan-2-one, ethyl acetate-D, Benzaldehyde, and pentan-2-one produced by maize at 18 h of storage can be used as early mildew biomarkers of *Aspergillus* infection in maize.

**Discussion:**

This study provided effective marker molecules for the development of an early warning and monitoring system for the degree of maize mildew in granaries.

## 1 Introduction

Maize, as an important food crop in China with uneven production and balanced consumption, is an important cash crop for national economic development. Chinese total maize output in 2022 is 261 million tons, accounting for about 39% of the country’s total grain output, of which about 10% is used for rations, 20% is used for industrial processing, and 70% is used for feed (Timm et al., 2022). Maize is very susceptible to mildew because of its nutrition, which not only directly affects the color, taste and processing of maize kernels (Niu and Xiang, 2018), but also results in a decline in the quality of maize. These changes are detrimental to storage stability and can lead to the loss of seed, food and feeding value of maize (Coradi et al., 2021). The essence of mildew is that microorganisms use organic matter in grain for catabolism. Microorganisms can carry out nutritional metabolic activities when they are in suitable environmental conditions, which in turn lead to mildew in grain (Zhang Y. et al., 2023). It has been reported that maize was frequently contaminated with fungal and produced mycotoxins, especially *Aspergillus*, which caused significant economic losses and health risks (Chandravarnan et al., 2022; Marshall et al., 2020). Therefore, early warning of *Aspergillus* risk is of great significance for the scientific control and security of agricultural products such as maize.

Thin-layer chromatography (TLC), high performance liquid chromatography (HPLC), Liquid chromatograph-mass spectrometer (LC-MS/MS) and enzyme-linked immunosorbent assay (ELISA) have been widely used for the detection mycotoxins in food and feed (Lu et al., 2024). However, these methods often suffer from complex steps, long operating times and poor detection sensitivity. With the continuous development of chromatography and spectroscopy techniques, the identification of mold in the food industry has shifted to the detection of growth metabolites such as mycotoxins and volatile organic compounds (VOCs) produced by mold (Gu et al., 2020). Microbial VOCs (MVOCs) are primary or secondary metabolic volatile compounds produced by bacteria and fungi during their growth and reproduction (Jiang et al., 2023; Chen et al., 2022). *Aspergillus flavus* (*A. flavus*) and *Aspergillus niger* (*A. niger*) consumed a variety of nutrients, produced a variety of metabolites, and released a variety of VOCs that affected the quality and safety of grains during the growth process. A new technique named gas chromatography-ion migration spectrometry (GC-IMS) monitoring system is commonly used in current research. Compared with electronic nose (E-nose) and gas chromatography-mass spectrometry (GC-MS), GC-IMS has the advantages of simple use, portable equipment, high sensitivity and no need for sample pretreatment (Parastar and Weller, 2024). At present, the application of this technique in food is mainly focused on wine, fruits and tea, but less research has been conducted in the field of maize mildew detection (Tang and Peng, 2024; Yang et al., 2024; Huang et al., 2023; Wang et al., 2020). Considering the above factors, GC-IMS was used to establish an effective method for the identification of VOCs in maize infected by *Aspergillus* at different growth stages. The infection process of *A. flavus* and *A. niger* was analyzed by constructing fingerprint spectra and heat maps, and the identification molecules of maize early mildew were found by combining principal component analysis (PCA) and cluster analysis, so as to explore the feasibility of the application of this method in the rapid assessment and early warning of the mildew degree of *Aspergillus*-infected maize.

## 2 Materials and methods

### 2.1 Materials

NK 718 maize kernels were harvested in Xingyang country, Zhengzhou city, Henan province, China, in 2023, and the initial moisture content was 13.5% (w/w). Potato dextrose agar (PDA) was acquired from Beijing Aobox Biotechnology Co., Ltd. (Beijing, China) and sodium hypochlorite (NaClO) was purchased from Shanghai Aladdin Biochemical Technology Co., Ltd. (Shanghai, China). All reagents used in this study were analytical-grade.

### 2.2 Isolation and identification of the dominant fungal strain from moldy maize kernels

The moisture content of maize was adjusted from 13.5 to 15% by referring to the method of Kinyungu et al. (2019) with minor modifications. A sample of 500 g of maize kernels with 15% moisture content was placed in a sterile transparent plastic bag and stored in an HWS constant temperature and humidity chamber (Ningbo Southeast Instrument Co., Ltd., Ningbo, Zhejiang, China) at 30°C until the sample became moldy. Then 25 g of moldy maize was mixed with 225 mL of sterile water and shaken in an HPY-92 Bed Temperature Incubator (Shanghai Yuejin Medical Equipment Co., Ltd., Shanghai, China) at 30°C and 150 rpm for 30 min. Subsequently, the suspension was evenly spread on PDA plates and incubated at 28°C for 3–5 days (Liu et al., 2022). Finally, single colonies were obtained by purification culture using the three-point inoculation method, from which strain MA and strain MB were screened for further experiments.

Strain MA and strain MB were inoculated on PDA plates and incubated at 28°C for 5 days. Then a drop of cotton blue staining solution was added to the slides, and the morphology of strains MA and MB were observed under the BM1000 Biological Microscope (Nanjing Jiangnan Yongxin Optical Co., Ltd., Nanjing, Jiangsu, China). The morphological characteristics of strain MA and strain MB were compared with GB/T 4789.16-2016. The DNA of fungal strains was extracted using the Magnetic Bead Method Fungal Genome Extraction Kit, and the 18S rRNA gene was amplified by PCR using the fungal universal primers ITS1 (5′-TCCGTAGGTGAACCTGCGG-3′) and ITS4 (5′-TCCTCCGCTTATTGATATATGC-3′) (Caligiore-Gei et al., 2023). The amplified products were detected by agarose gel electrophoresis and then sequenced by Beijing Liuhe Huada Gene Technology Co., Ltd. (Beijing, China). The 18S rRNA gene sequence results were uploaded to the National Center for Biotechnology Information (NCBI)-GenBank using Bankit Tool to obtain the NCBI login number. In addition, the 18S rRNA gene sequence of the strain was compared with the sequence in the NCBI database, and the sequence with higher homology was selected as the reference sequence. MEGA X software adopted neighbor-joining method to construct the phylogenetic tree (Kumar et al., 2018). Available online: https://www.ncbi.nlm.nih.gov/, accessed on 3 June 2024, and the accession numbers of strains MA and MB were PP843644 and PP843646, respectively.

### 2.3 Preparation of mildew maize samples

Maize kernels of uniform size with no insect infestation and no mechanical damage were selected, soaked in 0.3% sodium hypochlorite solution for 5 min, rinsed three times with sterile water, and then naturally ventilated at room temperature (Han et al., 2020). After drying the surface of the maize kernels, they were divided into two groups, each weighing about 250 g. The two groups of maize samples were immersed in suspensions of strain MA and strain MB spores with a concentration of about 10^6^–10^7^ spore/mL for 10 s, and then placed in a petri dish of sterile water agar medium, dried at room temperature (Majumdar et al., 2021). Subsequently, we placed them in an HWS constant temperature and humidity chamber at 30°C for cultivation and observation of contamination of maize by strain MA and strain MB (Zhang J. et al., 2023). Uncontaminated maize samples were used as control group. The samples were collected every 18 h. Among them, 5 g were stored in a 20 mL headspace vials in a refrigerator at −20°C for a total of four times, and three biological replicates were set up for each treatment.

### 2.4 Determination of total fungal colony counts in maize

The total fungal colony counts were determined by the plate count method (Zhang D. D. et al., 2024). Firstly, 25 g maize kernels were mixed with 225 mL sterile water and shaken well to make a fungal suspension with a concentration of 10^−1^, and then the fungal suspensions with dilution concentrations of 10^−2^ and 10^−3^ were prepared sequentially. After that, 100 μL of different dilutions of the fungal suspension were absorbed on PDA medium, evenly coated, and then placed in the incubator at 28°C for 3–5 days. Finally, the plates with colonies of 10–150 CFU were selected for counting, and the total number of fungal colonies was calculated. Three parallel experiments were performed for each dilution. For the sake of further analysis, the log of fungal spore quantity was calculated.

### 2.5 Determination of fungal spores in maize

The fungal spores in maize were determined by enumeration spores of fungi (Zhang et al., 2021). Firstly, 10 g maize kernels and 30 mL deionized water were placed in a 50 mL test tube, which was corked and shaken vigorously up and down for 1 min (about 120–150 times). Next, the mixture was homogenized and filtered with 300 mesh filter cloth and the filtrate was collected for subsequent determination. Finally, the fungal spores were counted using a hemocytometer (25 medium squares with V equal to 0.1 mm^3^). After the counting chamber of the hemocytometer was examined microscopically and free of dirt, a 20 mm × 20 mm coverslip was covered, and a drop of the filtrate was added to the plate with a rubber-tipped burette to ensure that no bubbles were generated. The fungal spores were counted for 30 s on a BM1000 biological microscope with an eyepiece of 10× and an objective of 10×. When the total number of spores in the 5 medium squares was less than 10, the number of spores in the 25 medium squares needed to be recorded. Two parallel counts were carried out for each sample, and the maximum of the two counts was taken as the experimental result. In addition, the maize samples were classified into four grades based on the results of fungal spores in storage, which were healthy (<1.0 × 10^5^), mild mildew (1.0 × 10^5^–9.9 × 10^5^), moderate mildew (1.0 × 10^6^–9.9 × 10^6^) and heavy mildew (≥1.0 × 10^7^). Three biological parallel experiments were performed for each sample.

### 2.6 GC-IMS analysis

The VOCs in maize were identified and quantitatively analyzed by GC-IMS flavor analyzer (FlavourSpec^®^ 1H1-00053, Dortmund, Germany) under different storage conditions. Referring to Kang et al. (2024) method and slightly modified, with 5 g maize kernels in a headspace vial, an injection needle temperature of 80°C, an incubation temperature of 60°C, an incubation time of 10 min, and 500 μL of sample injected by desorption in splitterless mode. A WAX capillary column (30 m, ID: 0.53 mm) at 50°C was used for chromatographic separation with nitrogen as carrier gas. Under isothermal conditions, carrier gas flow chromatography was used to separate the carrier gas flow rate from 2 mL/min for 2 min, increasing to 15 mL/min within 8 min, 100 mL/min within 10 min, and linearly increasing to 150 mL/min at 10 min. The total time for the analysis was 30 min (Li X. et al., 2019). Three biological parallel experiments were performed for each sample.

### 2.7 Statistical analysis

Statistical analysis and data visualization using SPSS Statistics 25 software, Origin (2022) software and Hiplot platform (Zhang D. D. et al., 2024). The Laboratory Analytical Viewer (LAV, G.A.S., Dortmund, Germany), three plug-ins (G.A.S., Dortmund, Germany) and GC-IMS Library Search were used to analyze the samples from different perspectives (Yang et al., 2019). Use the LAV analysis software that comes with the device Reporter, Gallery, Dynamic PCA plug-in and GC-IMS Library Search was used to construct difference maps of VOCs. Subsequently, two-dimensional qualitative analysis of the analytical sample materials was performed using the built-in NIST 2014 gas phase retention index database and the IMS migration time database of G.A.S. (Monedeiro et al., 2019). Meanwhile, PCA and heat maps were used to cluster maize samples (Pan et al., 2024).

## 3 Results

### 3.1 Dominant fungal strain of moldy maize kernels

**Figure 1:**
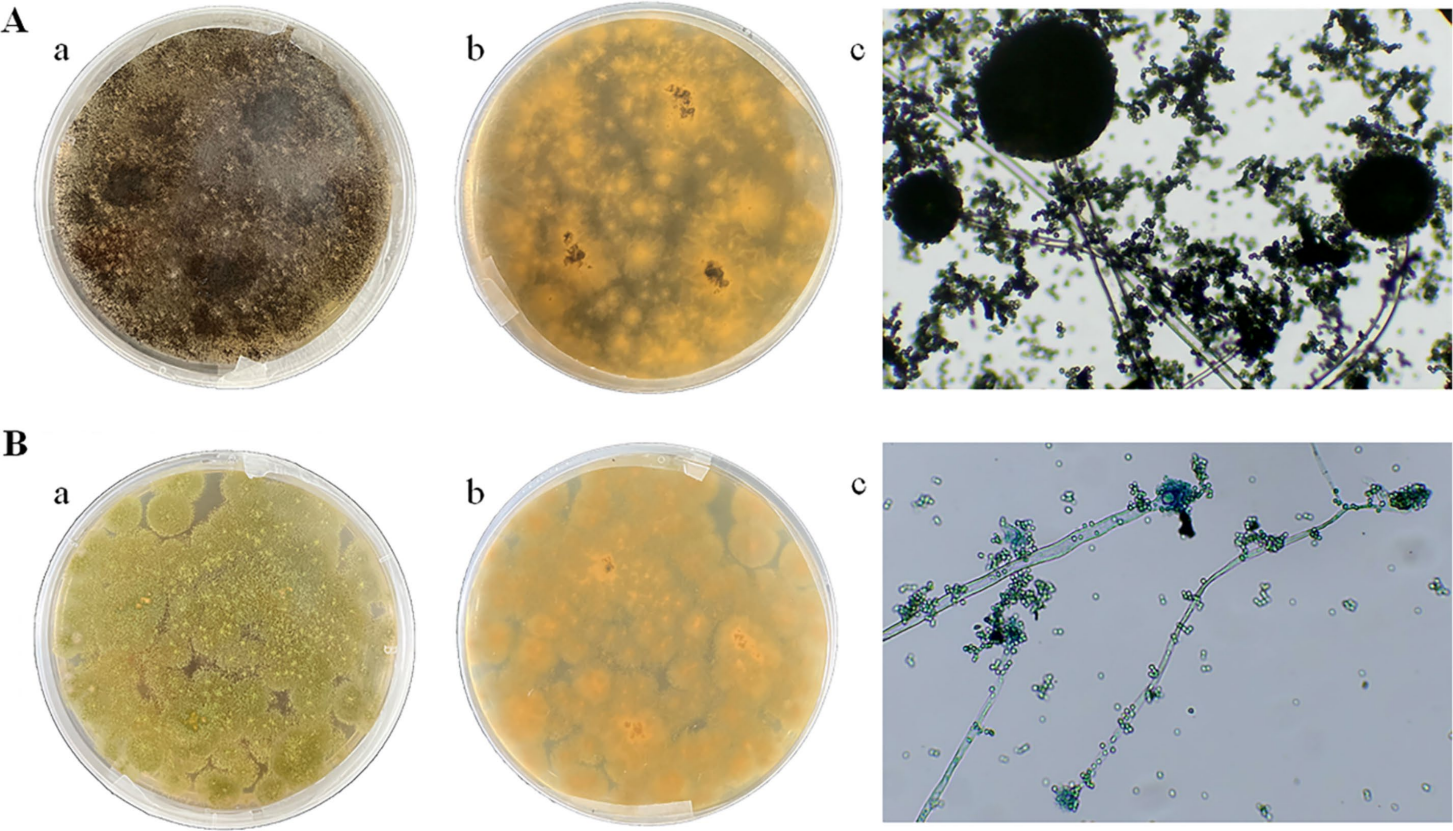
Morphological characteristics of strain MA and strain MB on PDA medium and under biological microscope. **(A)** Strain MA: **(a)** front side; **(b)** back side; **(c)** microscopic examination. **(B)** Strain MB: **(a)** front side; **(b)** back side; **(c)** microscopic examination.

**Figure 2:**
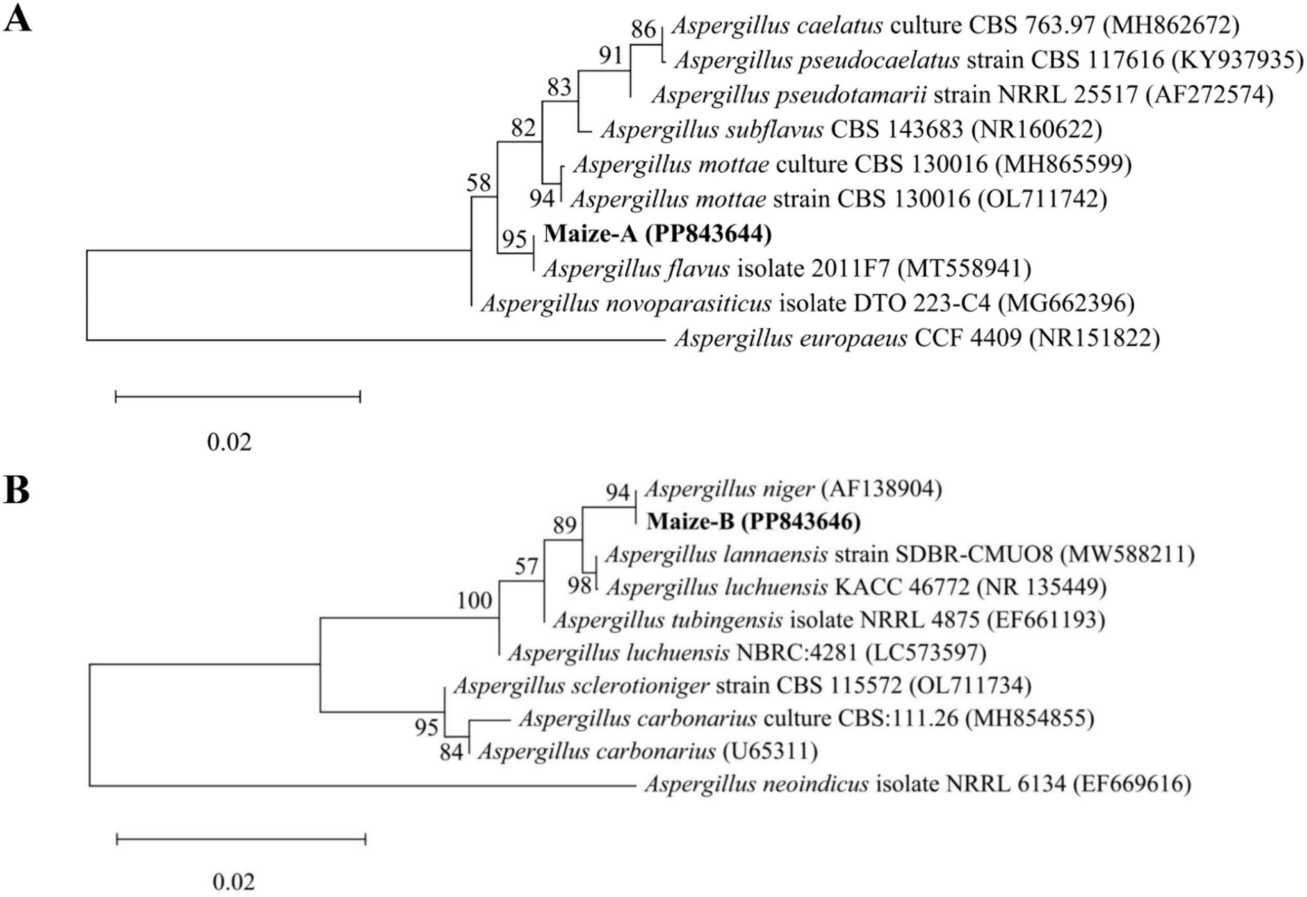
Phylogenetic tree of strain MA and strain MB and close reference strains based on 18S rRNA genes. **(A)** Phylogenetic tree of *Aspergillus flavus*. **(B)** Phylogenetic tree of *Aspergillus niger*.

Two dominant fungi, strain MA and strain MB, were isolated from moldy maize. The morphological characteristics of strain MA were shown in Figure 1A. The colony was slightly flocculent in texture, with a convex center and regular edges, and was yellowish green on the front side (Figure 1A,a). The middle of the dorsal surface of strain MA was light brown with no exudate (Figure 1A,b). The conidial peduncle was long, the terminal capsule was flask-shaped, and the conidia were mostly spherical and a few were oval (Figure 1A,c). The morphological characteristics of strain MB were shown in Figure 1B. Strain MB was fast growing, with flat colonies, loose texture, white at the beginning of the frontal surface and black velvet at the later stage (Figure 1B,a), while strain MB was yellow with no exudate on the back side (Figure 1B,b). The conidial peduncle was smooth and of variable length, the head acrosome was spherical, and the surface of the conidial was rough and black-brown spherical (Figure 1B,c). Phylogenetic tree was constructed based on 18S rRNA genes of strain MA and strain MB, closely related reference strains (Figure 2). On the base of point above, the dominant fungal strain MA was identified as *A. flavus*, and strain MB was identified as *A. niger*.

### 3.2 Total fungal colony counts in different moldy maize samples

**Figure 3:**
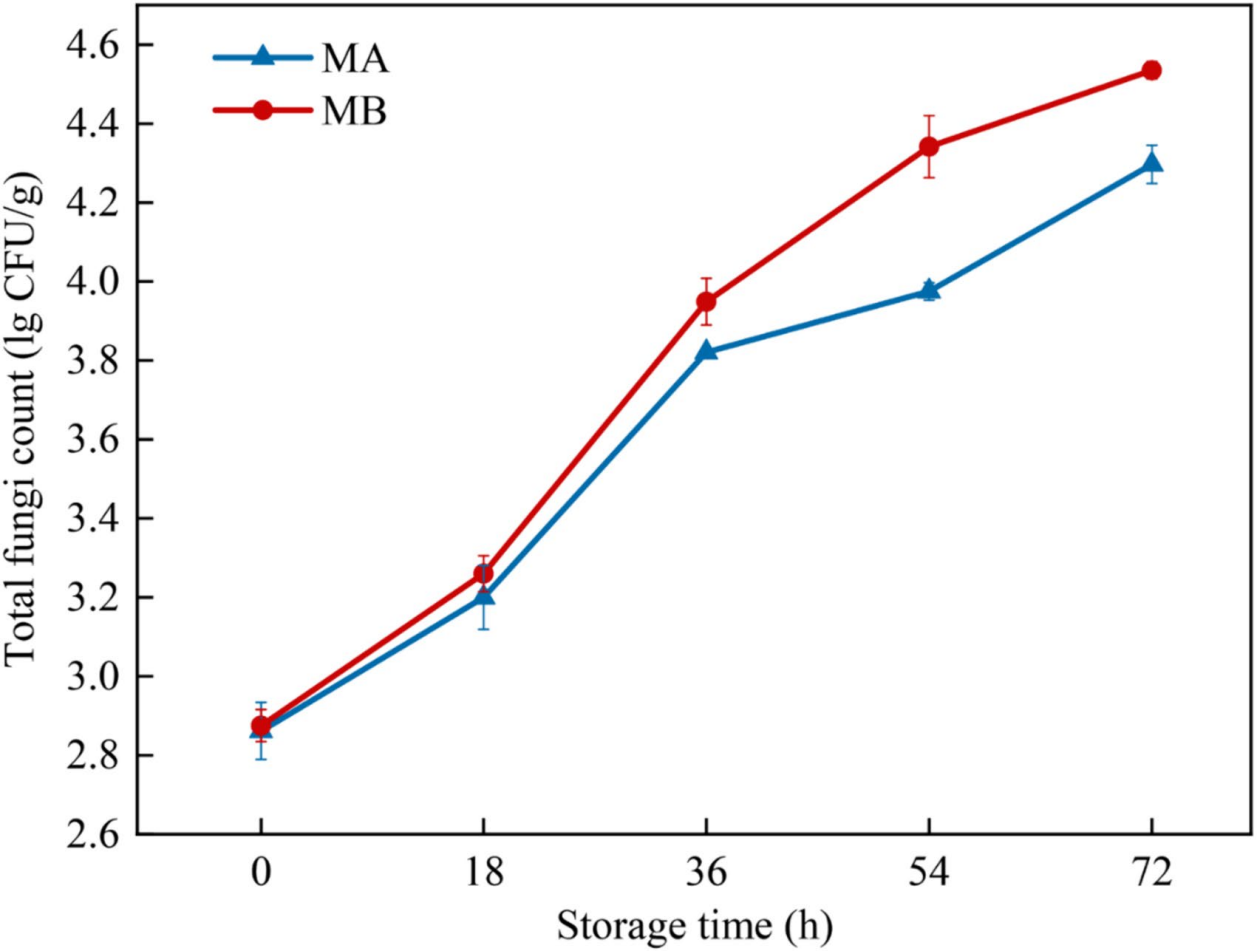
Total fungal colony counts in maize contaminated by dominant fungal strains MA and MB. The relative standard deviation of total fungi counts is represented with bars (*n* = 3).

The total fungal colony counts of maize infected with strains MA and MB were determined by the plate count method as shown in Figure 3. The results showed that with the extension of storage time, the total fungal colony counts of maize all showed an increasing trend. When stored for 0 h, the total colony counts of maize were all at a low level of about 2.8 lg CFU/g. During the period from 0 h to 18 h, the growth rate of the total colony counts of maize infected by the two fungi was relatively slow and in the delayed period, which was due to the fact that the nutrients in the kernels had not been completely absorbed (Guo et al., 2019). The total number of colonies of maize infected by the strain MA increased significantly during 18 h to 36 h, while the total number of colonies of maize infected by strain MB increased significantly during 18 h to 54 h, which was due to the fact that the fungi continuously absorbed nutrients in the early stage under suitable conditions and the cell division was slow. When the nutrients accumulated to a certain extent, the growth was rapid, which was in the logarithmic growth period. Then the growth of the total colony counts slowed down, and it was in the stable growth period. In addition, the total colony counts of maize infected by strain MB were consistently greater than those of strain MA-infected maize during the same storage time. The total colony count of strain MA-infected maize reached 4.2 lg CFU/g at 72 h, whereas the total colony count of strain MB-infected maize had reached 4.2 lg CFU/g at 54 h.

### 3.3 Fungal spores in different moldy maize samples

**Figure 4:**
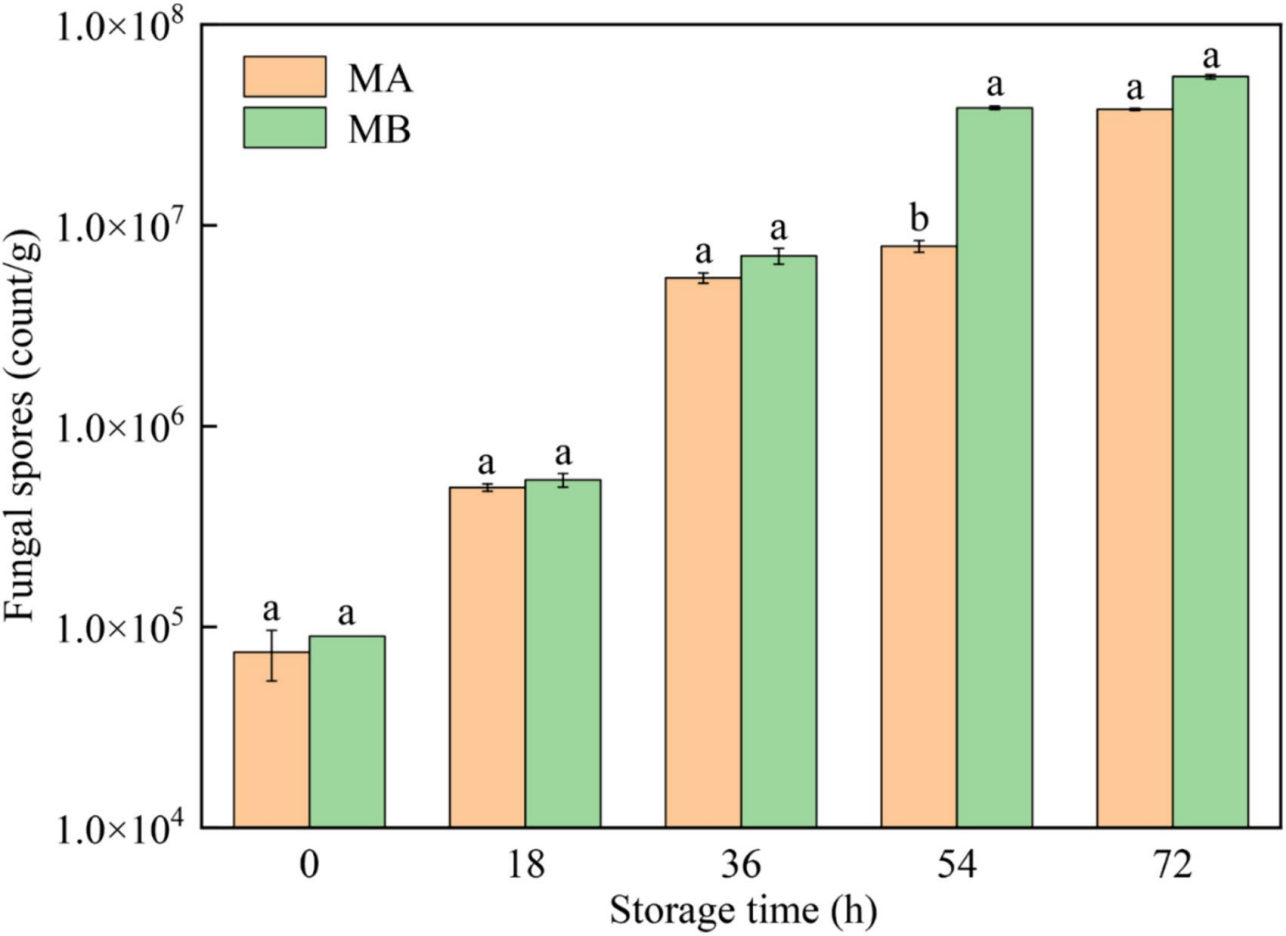
Fungal spores in maize contaminated by dominant fungal strains MA and MB. Different lowercase letters indicate significant differences (*p* < 0.05).

The results of fungal spores in different moldy maize samples were shown in Figure 4. It can be seen that with the growth of storage time, the number of fungal spores infecting maize by strains MA and MB both increased gradually, and the status of maize kernels gradually changed from healthy to heavy mildew (Figure 4). In the storage time of 0 h, the number of fungal spores in maize was less than 1.0 × 10^5^ count/g, which was in a healthy state. After storage for 18 h, the number of fungal spores gradually began to increase, and the maize kernels were mildly mildewed. When the maize was stored for 36 h, the number of fungal spores ranged from 1.0 × 10^6^ to 9.9 × 10^6^ count/g, which was in the moderate mildew state. In the storage time of 54 h, the maize infected by strain MA was still in a moderate mildew state, while the maize contaminated by strain MB was in a heavy mildew state, with the number of fungal spores greater than 1.0 × 10^7^ count/g. When stored for 72 h, all maize kernels were in a heavy mildew state. In addition, the number of fungal spores in maize contaminated by strain MB was greater than the number of fungal spores in maize infected by strain MA. Therefore, strain MB contaminated maize more severely than strain MA at the same time.

### 3.4 GC-IMS analysis of maize mold

**Figure 5:**
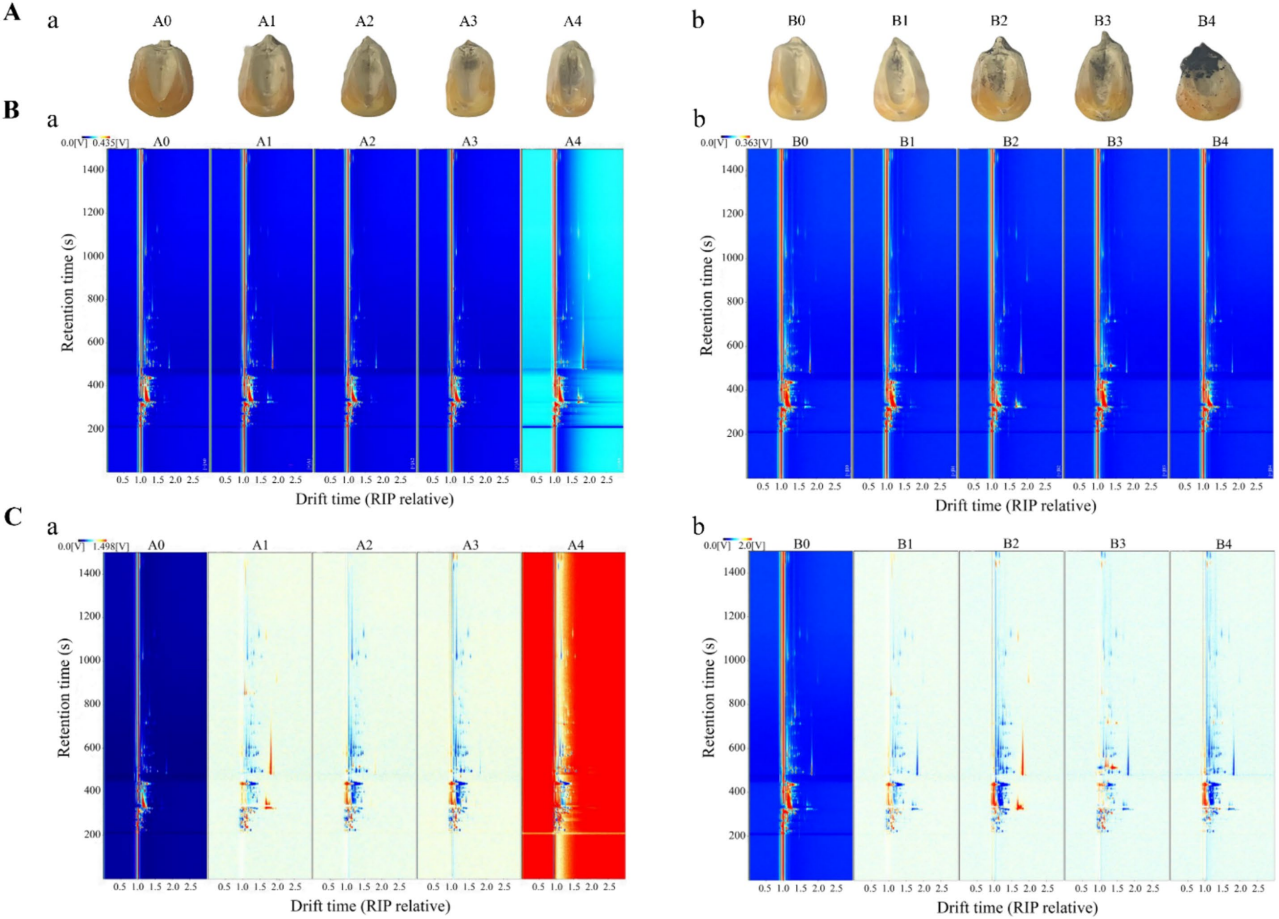
Maize samples and GC-IMS topography of maize samples at different storage periods. **(A)** Samples of maize at different mold growth stages (tested every 18 h): **(a)** *A. flavus*-contaminated maize samples; **(b)** *A. niger*-contaminated maize samples. **(B)** Top view of GC-IMS for VOCs in maize at different times (direct comparison): **(a)** *A. flavus*-contaminated maize samples; **(b)** *A. niger*-contaminated maize samples. **(C)** GC-IMS for VOCs of maize at different times (comparison of differences): **(a)** *A. flavus*-contaminated maize samples; **(b)** *A. niger*-contaminated maize samples. **A0, B0**: maize samples infected with *A. flavus* and *A. niger* for 0 h; **A1, B1**: maize samples infected with *A. flavus* and *A. niger* for 18 h; **A2, B2**: maize samples infected with *A. flavus* and *A. niger* for 36 h; **A3, B3**: maize samples infected with *A. flavus* and *A. niger* for 54 h; **A4, B4**: maize samples infected with *A. flavus* and *A. niger* for 72 h.

*A. flavus* and *A. niger* contamination of maize consumes many nutrients, produces various metabolites, and forms various VOCs, which affect the quality and flavor of maize. The VOCs produced by *A. flavus* and *A. niger* at different growth stages were analyzed by GC-IMS to explore the characteristic VOCs of *Aspergillus* at different infection stages in maize (Rahnavard et al., 2024). Figure 5A showed the mildew of maize kernels at different times after contamination by *A. flavus* and *A. niger*. A0 was the control group of *A. flavus*-contaminated maize, and A1-A4 were the samples collected at 18 h, 36 h, 54 h and 72 h after *A. flavus* contamination. B0 was the control group of *A. niger*-contaminated maize, and B1-B4 were the samples collected at 18 h, 36 h, 54 h and 72 h after *A. niger* contamination, respectively. Compared with the control group, the samples in groups A1 and B1 were mildly mildew, while samples in groups A2-A4 and B2-B4 were mildew more seriously. At the same time, it can be seen that the mildew phenomenon of *A. niger*-contaminated maize was more serious than that of *A. flavus*-contaminated maize, which may be related to the growth of mold. It was found in Figures 3, 4 that the total fungal colony counts and fungal spores in the maize infected by *A. niger* were larger than those in the maize infected by *A. flavus* at the same time, which was consistent with the phenomenon in this research.

To further investigate the changing pattern of VOCs in maize kernels at different growth stages of *Aspergillus* contamination, two-dimensional spectra of aromatic compounds in maize kernels were determined by GC-IMS as shown in Figure 5B. The total compounds in the sample headspace were shown by the entire spectrum, and each individual volatile compound was shown by the point to the right of the reactive ion peak (RIP). Substance signal intensities were shown by color, with white indicating low intensities and red indicating high intensities, and the darker the color, the higher the intensity. The results of Figure 5B showed that the retention time of most signals during the process of *A. flavus* and *A. niger* uncontaminated maize kernels appeared in the range of 200–1,200 s, with a drift time of 1.0–2.0 s.

In order to more obviously compare the flavor changes of maize kernels at different stages of *Aspergillus* infection, the spectra of A0 and B0 were selected as reference spectra, and the spectra of other infection times were deducted from the reference to calculate the differences between the reference spectra of A0 and B0 and the spectra of other groups (Figure 5C). The white background indicated that the two VOCs are consistent; the red represented that the concentration of the substance is higher than the reference; and the blue represented that the concentration of the substance is lower than the reference. Figure 5C showed that the concentration signals of most VOCs in the samples of the contaminated experimental groups A1–A4 and B1–B4 were much higher than the reference spectra of the A0 and B0 control groups, which indicated that VOCs produced in different growth stages of the maize contaminated by *A. flavus* and *A. niger* have great differences. After being contaminated by *Aspergillus*, some of the compounds that are easily decomposed or degraded disappeared from the signal or the signal intensity decreased. On the contrary, the enhancement of certain signal strengths also indicated that the concentration of certain compounds increased after infection.

### 3.5 Identification of VOCs in maize samples at different moldy growth stages

**Figure 6:**
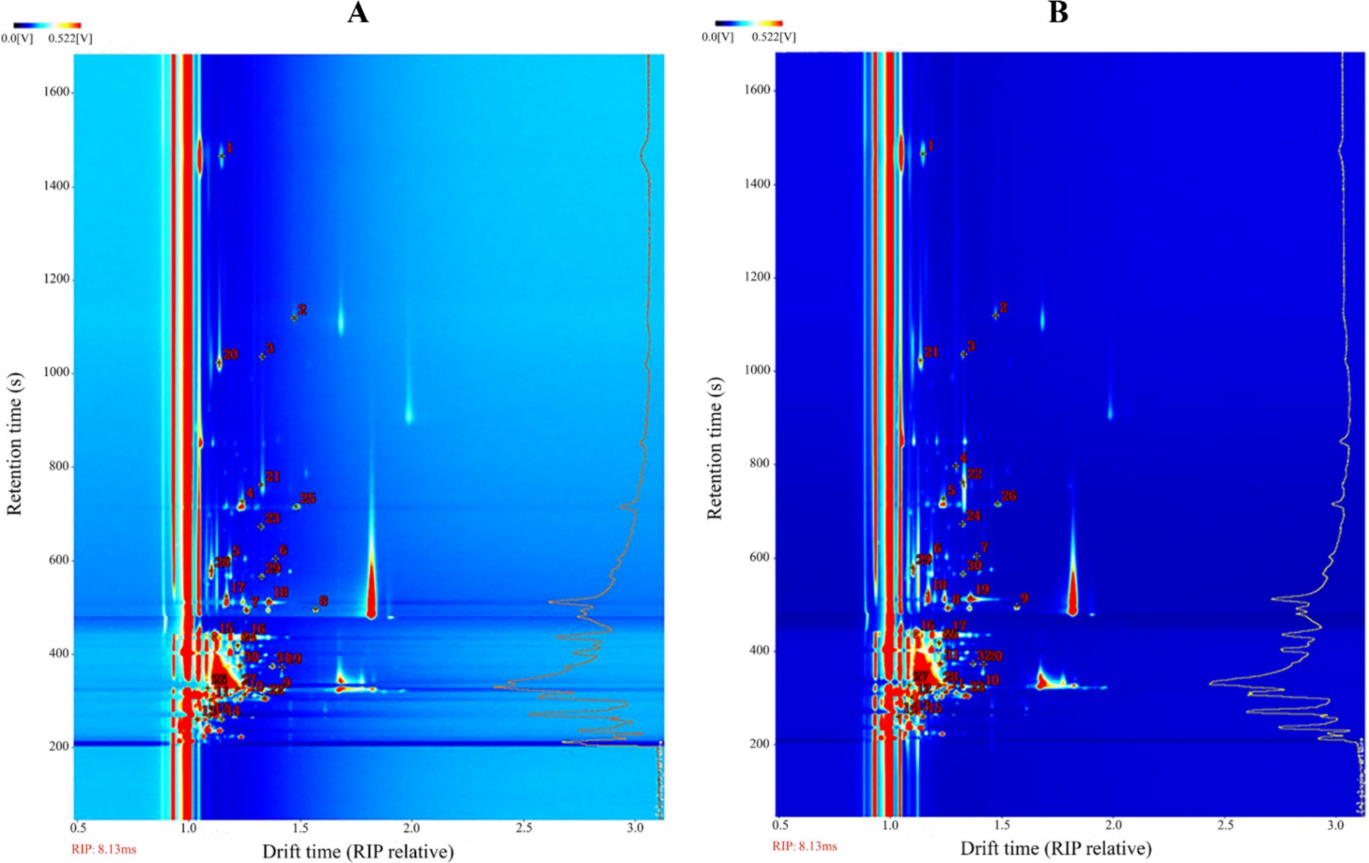
Ion mobility spectra of moldy maize. **(A)** *A. flavus*-contaminated maize samples. **(B)** *A. niger*-contaminated maize samples. The numbers indicate the identified VOCs.

The previous experiments found that the types and concentrations of VOCs in different stages of infection were significantly different, in which some compounds may also exist as a monomer, dimer or polymer to produce spots or multiple signals, resulting in differences in the spectrum, depending on the actual concentration and compound properties (Gu et al., 2021; Arroyo-Manzanares et al., 2018). The drift time and retention time of ion-mobility spectra (IMS) of VOCs were compared with reference organic compounds in the database by the Library Search software of GC-IMS. Overall, 31 compounds were identified from the maize kernels infected by *A. flavus* (Figure 6A and Table 1), and 32 compounds were identified from the maize kernels infected by *A. niger* (Figure 6B and Table 2), in which the suffixes M and D of the substance with the same name represented the monomers and dimers of the same compound, respectively.

**Table 1:** Information on identified compounds in *A. flavus* contaminated maize kernels.

No.	Compounds	CAS	Formula	MW	RI	Rt	Dt	Comment
1	Benzaldehyde	100-52-7	C_7_H_6_O	106.1	1506.6	1464.24	1.14983	
2	Nonanal	124-19-6	C_9_H_18_O	142.2	1,401	1118.877	1.47679	
3	1-Hexanol	111-27-3	C_6_H_14_O	102.2	1370.6	1035.614	1.33236	
4	3-Methylbutan-1-ol-M	123-51-3	C_5_H_12_O	88.1	1227.9	726.585	1.24232	Monomer
5	Butan-1-ol-M	71-36-3	C_4_H_10_O	74.1	1160.9	602.576	1.1799	Monomer
6	Butan-1-ol-D	71-36-3	C_4_H_10_O	74.1	1161.5	603.656	1.3922	Dimer
7	Hexanal-M	66-25-1	C_6_H_12_O	100.2	1098.8	493.02	1.26402	Monomer
8	Hexanal-D	66-25-1	C_6_H_12_O	100.2	1099.9	494.639	1.57045	Dimer
9	3-Methylbutanal	590-86-3	C_5_H_10_O	86.1	923.4	319.241	1.40822	
10	Butan-2-one	78-93-3	C_4_H_8_O	72.1	913.5	312.861	1.25379	
11	Ethyl acetate-M	141-78-6	C_4_H_8_O_2_	88.1	889.8	301.223	1.1012	Monomer
12	2-Methylpropanal	78-84-2	C_4_H_8_O	72.1	808.8	265.127	1.09618	
13	Propanal-M	123-38-6	C_3_H_6_O	58.1	795.2	259.506	1.04144	Monomer
14	Propanal-D	123-38-6	C_3_H_6_O	58.1	796.7	260.131	1.15026	Dimer
15	Propan-1-ol-M	71-23-8	C_3_H_8_O	60.1	1055.6	436.897	1.11842	Monomer
16	Propan-1-ol-D	71-23-8	C_3_H_8_O	60.1	1054.9	435.943	1.26182	Dimer
17	Isobutanol-M	78-83-1	C_4_H_10_O	74.1	1117.3	523.321	1.17167	Monomer
18	Isobutanol-D	78-83-1	C_4_H_10_O	74.1	1111.2	513.098	1.36328	Dimer
19	Valeraldehyde	110-62-3	C_5_H_10_O	86.1	996.3	370.833	1.42292	
20	2,6-Dimethyl pyrazine	108-50-9	C_6_H_8_N_2_	108.1	1365.8	1022.891	1.13984	
21	Ethyl hexanoate	123-66-0	C_8_H_16_O_2_	144.2	1247.7	762.06	1.32892	
22	Ethyl acetate-D	141-78-6	C_4_H_8_O_2_	88.1	899.9	306.044	1.34237	Dimer
23	Heptanal	111-71-7	C_7_H_14_O	114.2	1195.3	671.673	1.32757	
24	Alpha-pinene	80-56-8	C_10_H_16_	136.2	1039.6	417.814	1.22305	
25	3-Methyl-1-butanol-D	123-51-3	C_5_H_12_O	88.1	1221.3	715.041	1.48491	Dimer
26	Propan-2-ol-M	67-63-0	C_3_H_8_O	60.1	936	327.647	1.0871	Monomer
27	Propan-2-ol-D	67-63-0	C_3_H_8_O	60.1	935.8	327.489	1.22299	Dimer
28	Cyclopentanone-M	120-92-3	C_5_H_8_O	84.1	1147.8	577.486	1.10446	Monomer
29	Cyclopentanone-D	120-92-3	C_5_H_8_O	84.1	1,141	564.948	1.33116	Dimer
30	2-Methylpropyl acetate	110-19-0	C_6_H_12_O_2_	116.2	1000.9	375.161	1.2346	
31	Pentan-2-one	107-87-9	C_5_H_10_O	86.1	1,000	374.176	1.3781	

MW, molecular mass; RI, retention index; Rt, retention time; Dt, drift time.

**Table 2:** Information on identified compounds in *A. niger* contaminated maize kernels.

No.	Compounds	CAS	Formula	MW	RI	Rt	Dt	Comment
1	Benzaldehyde	100-52-7	C_7_H_6_O	106.1	1506.6	1464.24	1.14983	
2	Nonanal	124-19-6	C_9_H_18_O	142.2	1,401	1118.877	1.47679	
3	1-Hexanol	111-27-3	C_6_H_14_O	102.2	1370.6	1035.614	1.33236	
4	p-Cymene	99-87-6	C_10_H_14_	134.2	1266.1	796.537	1.2981	
5	3-Methylbutan-1-ol-M	123-51-3	C_5_H_12_O	88.1	1227.9	726.585	1.24232	Monomer
6	Butan-1-ol-M	71-36-3	C_4_H_10_O	74.1	1160.9	602.576	1.1799	Monomer
7	Butan-1-ol-D	71-36-3	C_4_H_10_O	74.1	1161.5	603.656	1.3922	Dimer
8	Hexanal-M	66-25-1	C_6_H_12_O	100.2	1098.8	493.02	1.26402	Monomer
9	Hexanal-D	66-25-1	C_6_H_12_O	100.2	1099.9	494.639	1.57045	
10	3-Methylbutanal	590-86-3	C_5_H_10_O	86.1	923.4	319.241	1.40822	
11	Butan-2-one	78-93-3	C_4_H_8_O	72.1	913.5	312.861	1.25379	
12	Ethyl acetate-M	141-78-6	C_4_H_8_O_2_	88.1	889.8	301.223	1.1012	Monomer
13	2-Methylpropanal	78-84-2	C_4_H_8_O	72.1	808.8	265.127	1.09618	
14	Propanal-M	123-38-6	C_3_H_6_O	58.1	795.2	259.506	1.04144	Monomer
15	Propanal-D	123-38-6	C_3_H_6_O	58.1	796.7	260.131	1.15026	Dimer
16	Propan-1-ol-M	71-23-8	C_3_H_8_O	60.1	1055.6	436.897	1.11842	Monomer
17	Propan-1-ol-D	71-23-8	C_3_H_8_O	60.1	1054.9	435.943	1.26182	Dimer
18	Isobutanol-M	78-83-1	C_4_H_10_O	74.1	1117.3	523.321	1.17167	Monomer
19	Isobutanol-D	78-83-1	C_4_H_10_O	74.1	1111.2	513.098	1.36328	Dimer
20	Valeraldehyde	110-62-3	C_5_H_10_O	86.1	996.3	370.833	1.42292	
21	2,6-Dimethyl pyrazine	108-50-9	C_6_H_8_N_2_	108.1	1365.8	1022.891	1.13984	
22	Ethyl hexanoate	123-66-0	C_8_H_16_O_2_	144.2	1247.7	762.06	1.32892	
23	Ethyl acetate-D	141-78-6	C_4_H_8_O_2_	88.1	899.9	306.044	1.34237	Dimer
24	Heptanal	111-71-7	C_7_H_14_O	114.2	1195.3	671.673	1.32757	
25	Alpha-pinene	80-56-8	C_10_H_16_	136.2	1039.6	417.814	1.22305	
26	3-Methyl-1-butanol-D	123-51-3	C_5_H_12_O	88.1	1221.3	715.041	1.48491	Dimer
27	Propan-2-ol-M	67-63-0	C_3_H_8_O	60.1	936	327.647	1.0871	Monomer
28	Propan-2-ol-D	67-63-0	C_3_H_8_O	60.1	935.8	327.489	1.22299	Dimer
29	Cyclopentanone-M	120-92-3	C_5_H_8_O	84.1	1147.8	577.486	1.10446	Monomer
30	Cyclopentanone-D	120-92-3	C_5_H_8_O	84.1	1,141	564.948	1.33116	Dimer
31	2-Methylpropyl acetate	110-19-0	C_6_H_12_O_2_	116.2	999	373.149	1.22439	
32	Pentan-2-one	107-87-9	C_5_H_10_O	86.1	999	373.149	1.37877	

MW, molecular mass; RI, retention index; Rt, retention time; Dt, drift time.

### 3.6 Fingerprints and heat map cluster analysis of VOCs in different moldy maize samples

In order to more directly observe the variation rules and relative content changes of VOCs in the process of *Aspergillus* infection of maize, the fingerprint spectrum of VOCs was plotted with the help of Gallery Plot plug-in, and the differences of VOCs among different samples were compared intuitively. Furthermore, the signal intensity of each aromatic compound indicated its concentration level (Chen et al., 2021).

**Figure 7:**
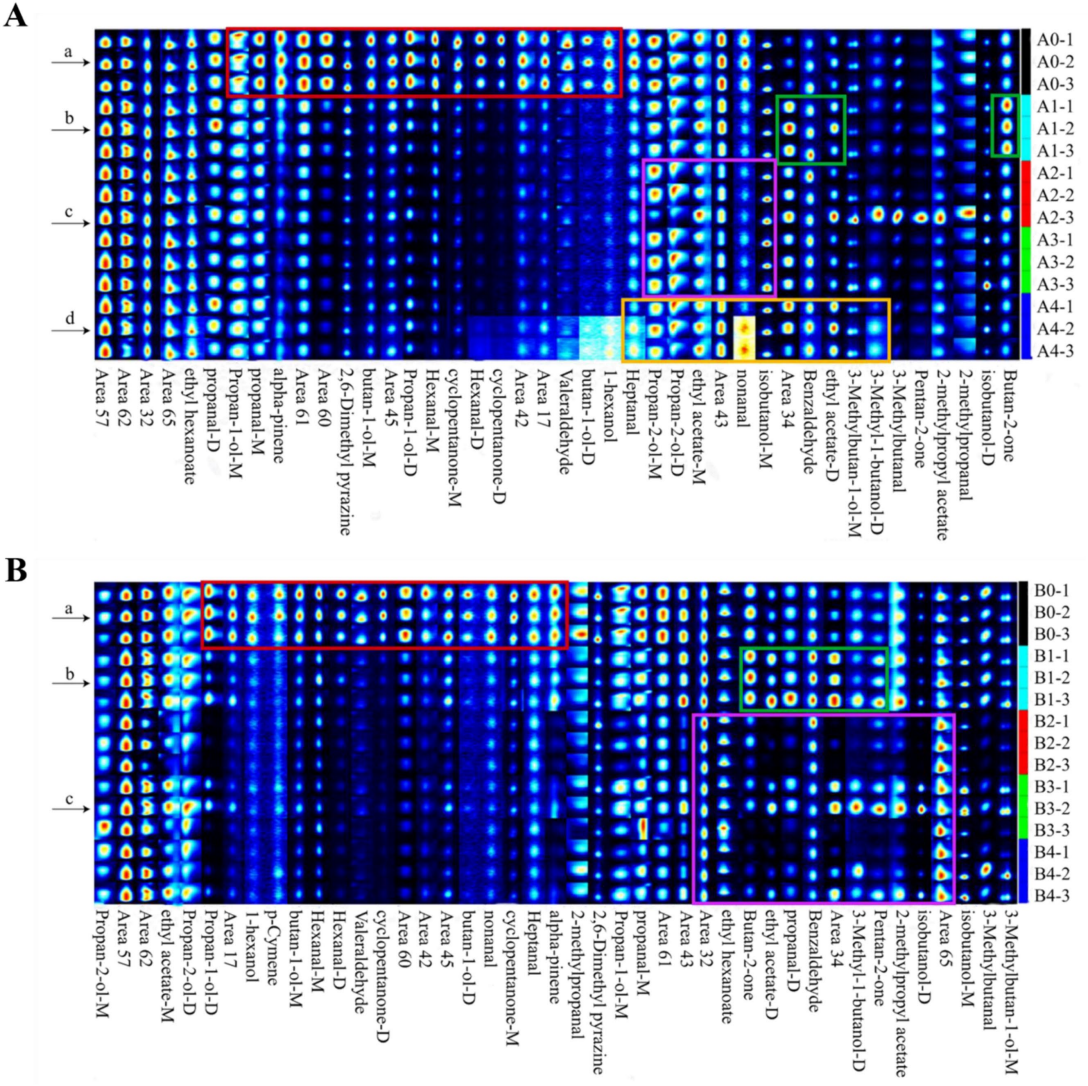
Comparison of the fingerprints of VOCs in uninoculated and inoculated *Aspergillus* samples determined by GC-IMS. **(A)** *A. flavus*-contaminated maize samples. **(B)** *A. niger*-contaminated maize samples.

Figure 7A showed the differential fingerprints of VOC species and concentrations in *A. flavus*-contaminated maize samples. As can be seen from Figure 7A, the signal intensity of VOCs such as 1-hexanol, butan-1-ol-D, valeraldehyde, cyclopentanone-D, hexana-M, propan-1-ol-D, 2, 6-dimethylpyrazine and alpha-pinene was very strong in the 0 h samples from region a. However, benzaldehyde, ethyl acetate-M, butan-2-one and other substances were almost non-existent in region a, and the strongest signal was found in the 18 h samples from region b. The concentrations of VOCs in regions c and d, such as 3-methyl-1-butanol-D, isobutanol-M, nonanal, propan-2-ol-M and heptanal, were higher than those in samples A2, A3, and A4 in the middle and late stages of the contamination, which were the main VOCs in the middle and late stages of maize mildew. Figure 7B showed the differential fingerprints of VOC species and concentrations in maize samples contaminated by *A. niger*. The signal intensities of VOCs such as alpha-pinene, heptanal, cyclopentanone-M, nonanal, butan-1-ol-D, valeraldehyde, hexanal-M, hexanal-D, p-cymene and others in the B0 sample of region a were much higher than those in the group of *A. niger*-contaminated samples. pentan-2-one, benzaldehyde, propanal-D, ethyl acetate-D and butan-2-one in region b were mainly presented in group B1 samples at the early stage of mildew. VOCs in region c such as ethyl hexanoate, isobutanol-D and 2-methylpropyl acetate appeared at 36–72 h in the middle and late stages of infection, and the signal intensity of these substances increased with the increase in *A. niger* infection time. The results showed that there were significant differences in VOCs at different infection stages in the experimental group, which provided a possibility for further monitoring of maize mildew.

**Figure 8:**
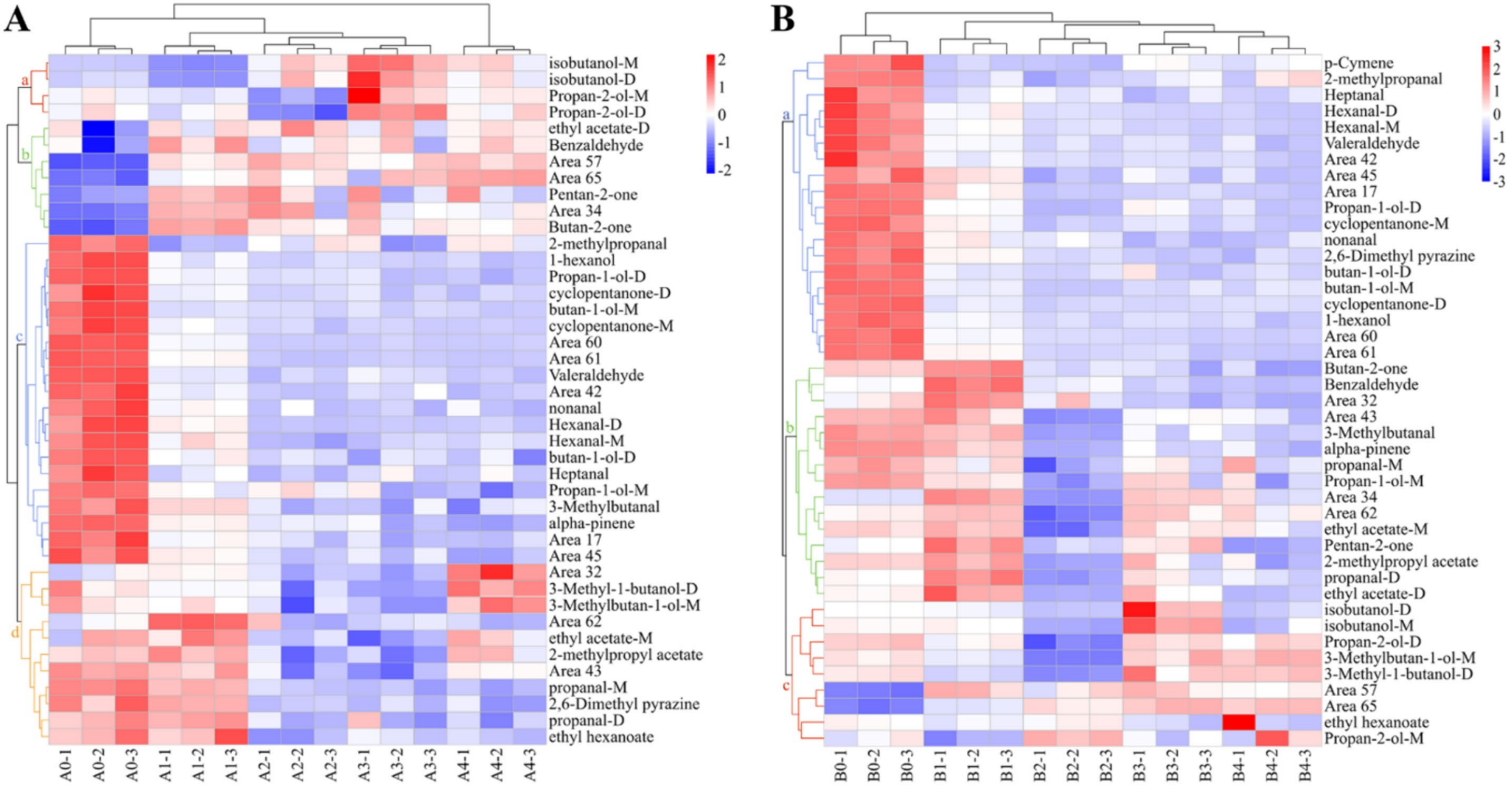
Heat map and cluster analysis of maize samples with different degrees of infection. **(A)** *A. flavus*-contaminated maize samples. **(B)** *A. niger*-contaminated maize samples.

Cluster analysis of the data using heat maps was performed to further understand the differences in VOCs in *Aspergillus* contaminated maize kernels at different mold growth stages (Figure 8). Based on the vertical direction of the heat map, all samples were divided into three groups: the control group (A0, B0), the pre-mold stage (A1, B1) and the middle and late mold stages (A2–A4, B2–B4). The cluster analysis of VOCs in the *A. flavus*-contaminated maize kernels (Figure 8A) divided the substances in the samples into four categories: a, b, c and d. Among them, Group c was mainly found in the control group A0, the VOCs of Group b were mainly produced in the pre-mold stage A1, and the Groups a and d were mainly produced in the middle and late stages of mold A2–A4. The VOCs cluster analysis diagram of maize samples contaminated by *A. niger* (Figure 8B) classified the substances into three categories: a, b and c. The VOCs of Group a mainly existed in the control group B0, Group b was mainly produced in the pre-mold stage B1, and Group c was mainly produced in the middle and late stages B2–B4. Therefore, in order to achieve early monitoring and early warning, we focused on the detection targets of the compounds in Group b (Figure 8). The signals of benzaldehyde, pentan-2-one, butan-2-one and ethyl acetate in Group b were the strongest in the early stage of mildew. What’s more, it could be seen that the signals gradually weakened with the increase in the growth time of *A. flavus* and *A. niger*. The results showed that the sample group and the VOCs group set up in the experiment were well representative.

### 3.7 PCA analysis of VOCs in different moldy maize samples

**Figure 9:**
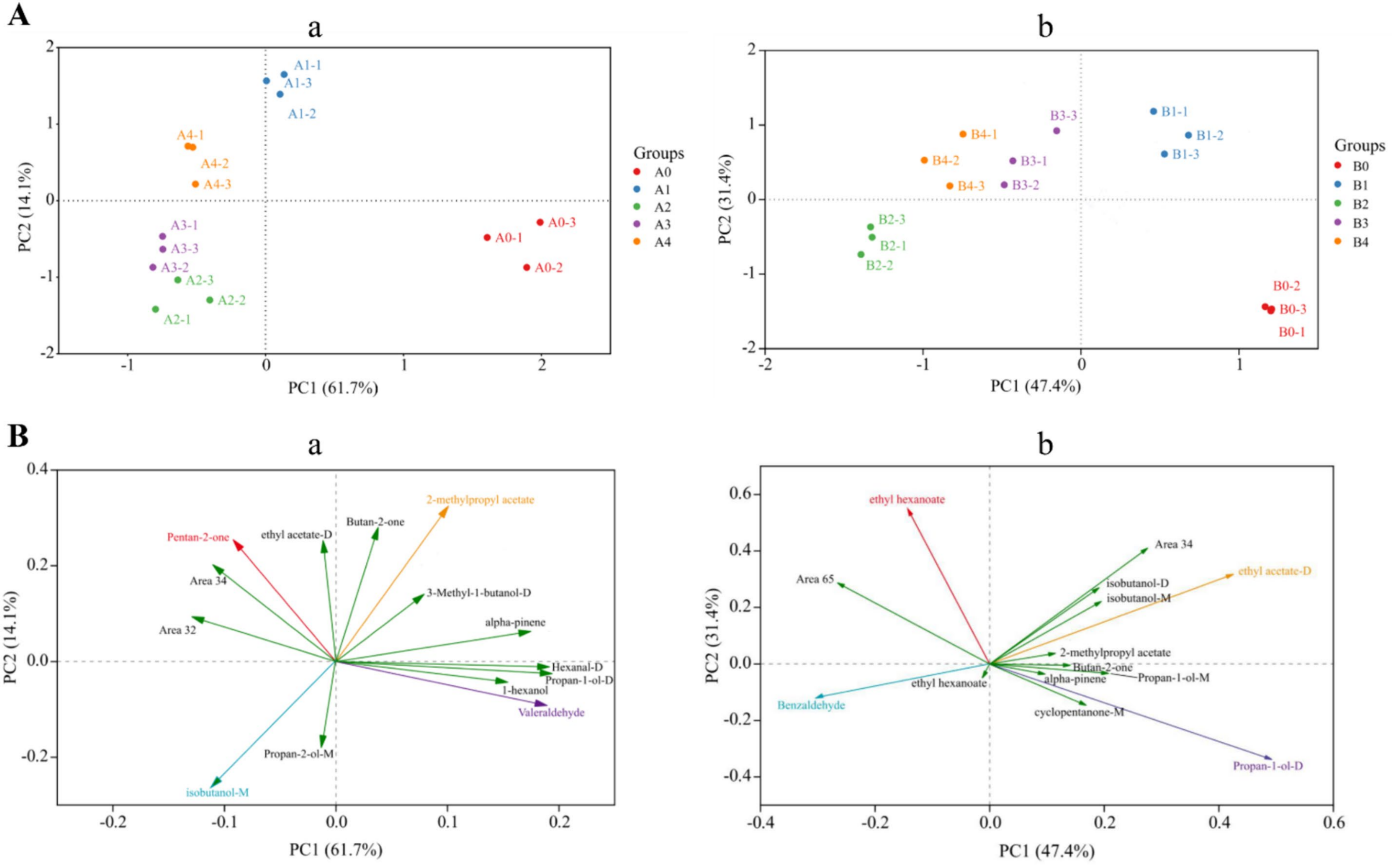
PCA analysis based on signal strength of maize samples. **(A)** Plot of the top two principal component scores: **(a)** *A. flavus*-contaminated maize samples; **(b)** *A. niger*-contaminated maize samples. **(B)** Plot of principal component loadings: **(a)** *A. flavus*-contaminated maize samples; **(b)** *A. niger*-contaminated maize samples.

Principal component analysis (PCA) is one of the most widely used data dimensionality reduction algorithms, the basic idea of which is to try to extract the main information of the data, and then discard the redundant information, so as to achieve the purpose of compression, while retaining the original data information to the greatest extent (Cavanna et al., 2019). Figure 9 showed the PCA data obtained from the analysis of the entire data of VOCs produced by *Aspergillus*-contaminated maize, which is divided into two parts: 1 and 2. Principal component 1 (PC1) accounted for 61.7% of the variance in the data, and PC2 accounted for 14.1% of the variance in Figure 9A,a. The results of the PCA were divided into four different regions, where region a included the A0 control group, region b included the pre-mold stage A1, region c included the middle stage of A2 and A3 mildew, and region d included the late stage of A4 mildew. In Figure 9A,b, PC1 and PC2 accounted for 47.4 and 31.4% of the variance in the data, respectively. The results of the PCA were divided into four different regions, where region a included the B0 control group, region b included the pre-mold stage B1, region c included the middle stage of B2 mildew, and region d included the late stage of B3 and B4 mildew. The PCA analysis of *A. flavus* and *A. niger* contaminated maize kernels showed a high degree of similarity in VOCs concentrations between samples from regions c and d, but significant differences between region b and the other regions. The results of the PCA were similar to the results of the previous cluster analysis heat maps.

Screening was performed using principal component loading plots to identify the VOCs that contributed the most to different principal component clusters. The direction and length of the vectors therein indicate the extent to which the variables contribute to the two principal components. It can be seen from Figure 9B,a that Valeraldehyde was positively correlated with the control group of *A. flavus*-contaminated maize samples, and 2-methylpropyl acetate was positively correlated with the samples at the early stage of aflatoxin growth. Then, for samples in the middle and late stages of mold growth, pentan-2-one and isobutanol-M were significantly positively correlated with the maize samples. In addition, the loading diagram of VOCs in maize samples contaminated by *A. niger* (Figure 9B,b) showed that propan-1-ol-D was positively correlated with the control group of maize samples, whereas ethyl acetate-D, which is the main characteristic flavor of B1, was the main cause of PC1.

**Figure 10:**
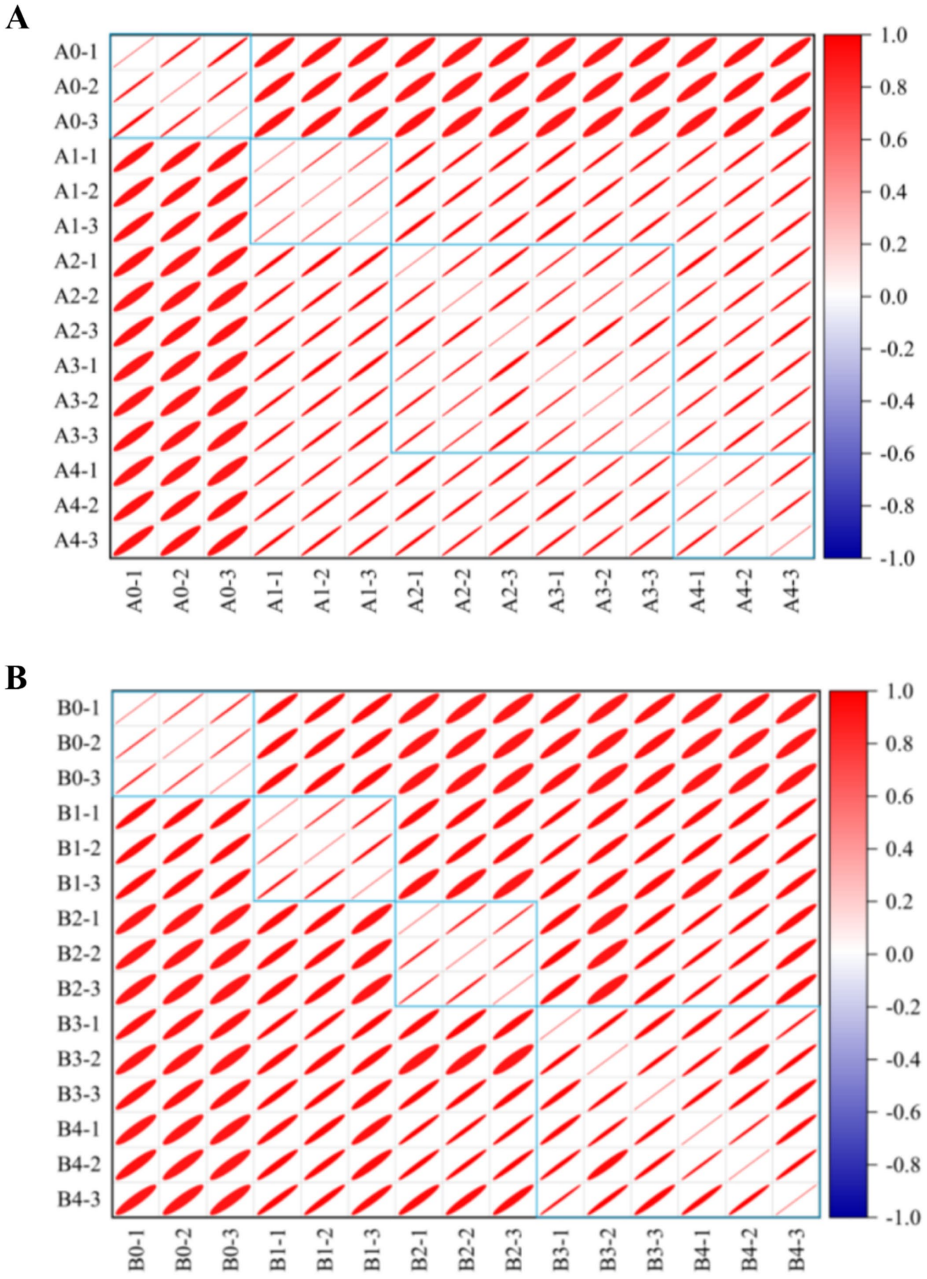
Similarity analysis of different groups in maize samples. **(A)** *A. flavus*-contaminated maize samples. **(B)** *A. niger*-contaminated maize samples. The colors in the similarity analysis between VOCs represent the degree of similarity from no correlation (dark blue) to high correlation (dark red). The area of the ellipse from small to large represents the correlation between VOCs from strong to weak.

The similarity between VOCs was analyzed and plotted in Figure 10, which showed that the differences between *A. flavus* and *A. niger* in maize samples in the pre-growth stage (A1, B1) and in the late growth stages (A2–A4, B2–B4) were large. The maize samples of A1 and B1 in the pre-mold stage could be separated from A0 and B0 in the control group. This finding is consistent with fingerprints and heat maps. This result provided a possibility for early monitoring and early warning of *Aspergillus* contamination in maize.

## 4 Discussion

As one of the major food crops in China, maize is an important source of raw materials and feed for the chemical and food industries, whose total output is second only to rice and wheat (Zou et al., 2024). However, maize is very susceptible to mold contamination during growth, harvesting, transportation and processing (Bocate et al., 2021), especially *Aspergillus*. Brito et al. (2022) pointed out that *Aspergillus* in maize stored in silo-bags mainly consisted of *A. flavus*, *A. fumigatus* and *A. niger*, with the highest incidence being *A. flavus* (61.75%). In this study, the predominant strains of mildew were selected from moldy maize, and *A. flavus* and *A. niger* were identified by morphological characterization and 18S rRNA gene sequence analysis (Figures 1, 2). Moldy maize not only leads to lower quality and economic losses, but can even pose a health risk to humans. *A. flavus* has been reported to produce aflatoxins such as AFB1 and AFB2, and *A. niger* produced B-group fumonitins, which were known to have deleterious effects such as teratogenicity, carcinogenesis and nephrotoxicity (Norlia et al., 2019; Zhang D. et al., 2024). Therefore, monitoring and controlling the quality of maize during storage based on *A. flavus* and *A. niger* is one of the main objectives of maize storage. Paolesse et al. (2006) used GC-MS to analyze maize infected with mold, and the experimental results showed that the VOCs of moldy maize included 3-methyl-1-butanol, 1-hexene, etc. Karlshøj et al. (2007) showed that VOCs such as ethanol, 2-methyl-1-propanol, 3-methyl-1-butanol, acetone and 2-butanone were released during the synthesis of certain mycotoxins, and that fungal contamination can be predicted by E-nose monitoring. However, the sensors used are costly and short-lived. GC-IMS, as a new technique, has the advantages of simple sample preparation, easy operation, high sensitivity, fast analytical speed, and the ability to even detect trace VOCs in a short period of time (Li M. et al., 2019).

Using GC-IMS technique in this study, 31 and 32 VOCs were identified from *A. flavus* and *A. niger*-contaminated maize, respectively. Furthermore, the results of PCA analysis and cluster analysis clearly demonstrated that different samples occupied distinct and discriminative spaces, indicated good representativeness in the preparation of moldy samples, which offered a viable approach for leveraging differences in flavor substance abundance and composition at various infection stages to assess and differentiate safety issues related to maize. Combined with GC-IMS fingerprinting, cluster analysis and PCA analysis, it was found that the maize contaminated by *A. flavus* and *A. niger* all began to mold at 18 h and entered the early stage of mold. In addition, the results of the determination of the total colony counts of moldy maize can be seen in Figure 3, which showed that the total colony counts of *A. flavus* and *A. niger*-infected maize were 3.20 lg CFU/g and 3.26 lg CFU/g, respectively, when stored for 18 h. Omobowale et al. (2016) suggested that when the total colony counts exceeded 1,000 CFU/g (i.e., 3 lg CFU/g), maize would become mildewed and be at an unsafe level for human consumption. As depicted in Figure 4, the maize contaminated by *A. flavus* and *A. niger* was healthy at 0 h of storage, while the fungal spore counts were between 1.0 × 10^5^ and 9.9 × 10^5^ at 18 h of storage, at which time the maize kernels underwent mild mold and were in the early stages of mold. Therefore, the determination of GC-IMS, total fungal colony counts and the number of fungal spores in maize can be concluded that the *Aspergillus*-infected maize kernels entered the early stages of mold at 18 h of storage.

This study focused on the analysis of potential biomarkers for the early stages of *A. flavus* and *A. niger* contamination of maize, the abundances of Butan-2-one, ethyl acetate-D, benzaldehyde and pentan-2-one were strongest in the pre-mold stage (18 h), while the concentration signal diminished in the middle and late stages of molding (36–72 h). Li et al. (2021) investigated the VOCs changes in maize mold using GC-IMS technique and found that ethyl acetate-D and 3-hydroxybutan-2-one-D could be used as early warning molecules for maize infection by *A. flavus*. This is in general consistent with our findings. Wang et al. (2021) analyzed potential biomarkers at different stages of *A. flavus*-contaminated walnuts and found that ethyl acetate-D and cyclohexanone had the strongest signals at the pre-mold stage, and the result suggested that the establishment of a suitable gas sensor to monitor the VOCs content at different stages of *A. flavus* infection in walnuts was feasible. This study provides effective monitoring target molecules for the development of gas monitoring sensors for early mold contamination of maize under storage conditions, and a real-time online monitoring and early warning system for maize mold under storage conditions can be designed based on these labeled molecules (Li et al., 2021).

In this study, a simple, efficient and specific method for evaluating the VOCs of maize samples infected by *Aspergillus* was established. Using GC-IMS for detection, no additional processing process is required during sample sampling, which shortens the analysis time. Collecting VOCs in a storage environment can efficiently and quickly monitor agricultural products, which provides a new idea for early warning and monitoring of maize mildew, and has great application prospects.

## 5 Conclusion

In this study, we screened maize mold dominant strains from moldy maize and identified *A. flavus* and *A. niger* by morphological and 18S rRNA gene sequence analysis. These two kinds of fungi were used to infect fresh maize and make it moldy, respectively. Then, using the GC-IMS technique, 31 and 32 VOCs were identified from *A. flavus* and *A. niger*-contaminated maize without sample pretreatment. The results showed that the abundance and composition of flavor substances in maize samples varied greatly at different growth stages of mold. Differences in the composition, abundance and composition of VOCs in stored maize contaminated with *Aspergillus* were evaluated by fingerprints, cluster analysis and PCA analysis. Combining the total fungal colony counts and fungal spores of *A. flavus* and *A. niger*-infected maize as well as the results of GC-IMS revealed that the maize entered the early stage of mold at 18 h. In conclusion, it can be seen that butan-2-one, ethyl acetate-D, benzaldehyde and pentan-2-one were strongest when stored for 18 h in the pre-mold stage of fungal infection, and the concentration signals decreased with the increasing growth time. Therefore, it was feasible that GC-IMS could be used for early warning of mold in maize.

## Data Availability

The datasets presented in this study can be found in online repositories. The names of the repository/repositories and accession number(s) can be found in the article/supplementary material.
